# Prevalence of Endocrinopathies in a Cohort of Patients with Rett Syndrome: A Two-Center Observational Study

**DOI:** 10.3390/genes15030287

**Published:** 2024-02-24

**Authors:** Giorgia Pepe, Roberto Coco, Domenico Corica, Gabriella Di Rosa, Filip Bossowski, Magdalena Skorupska, Tommaso Aversa, Stefano Stagi, Malgorzata Wasniewska

**Affiliations:** 1 Pediatric Unit, Department of Human Pathology of Adulthood and Childhood, University of Messina, Via Consolare Valeria 1, 98125 Messina, Italy; giopepe@unime.it (G.P.); cocoroberto93@gmail.com (R.C.); domenico.corica@unime.it (D.C.); bossowski.filip@gmail.com (F.B.); magdalenaskorupska18@gmail.com (M.S.); taversa@unime.it (T.A.); 2 Child Neuropsychiatric Unit, Department of Human Pathology of Adulthood and Childhood, University of Messina, 98128 Messina, Italy; gabriella.dirosa@unime.it; 3 Department of Health Sciences, University of Florence, 50139 Florence, Italy; stefano.stagi@unifi.it; 4 Meyer Children Hospital IRCCS, 50139 Florence, Italy

**Keywords:** Rett syndrome, endocrinopathy, MeCP2 deletion, CDKL5 deletion, epilepsy

## Abstract

Systematic data on endocrinopathies in Rett syndrome (RTT) patients remain limited and inconclusive. The aim of this retrospective observational two-center study was to assess the prevalence of endocrinopathies in a pediatric population of RTT patients. A total of 51 Caucasian patients (47 girls, 4 boys) with a genetically confirmed diagnosis of RTT were enrolled (mean age 9.65 ± 5.9 years). The patients were referred from the Rett Center of two Italian Hospitals for endocrinological evaluation. All the study population underwent clinical and auxological assessments and hormonal workups. *MeCP2* mutations were detected in 38 cases (74.5%), *CDKL5* deletions in 11 (21.6%), and *FOXG1* mutations in 2 (3.9%). Overall, 40 patients were treated with anti-seizure medications. The most frequent endocrinological finding was short stature (47%), followed by menstrual cycle abnormalities (46.2%), weight disorders (45.1%), low bone mineral density (19.6%), hyperprolactinemia (13.7%) and thyroid disorders (9.8%). In the entire study population, endocrinopathies were significantly more frequent in patients with *MeCP2* mutations (*p* = 0.0005), and epilepsy was more frequent in *CDKL5* deletions (*p* = 0.02). In conclusion, our data highlighted that endocrinopathies are not rare in RTT, especially in patients with *MeCP2* deletions. Therefore, in the context of a multidisciplinary approach, endocrinological evaluation should be recommended for RTT patients.

## 1. Introduction

Rett syndrome (RTT) is a severe, progressive neurodevelopment disorder that has been identified almost exclusively in girls [1]. It is an X-linked syndrome, often characterized by loss-of-function mutations in the ubiquitously expressed *MeCP2* (methyl CpG binding protein 2) gene [2]. *MeCP2* protein is an essential transcriptional regulator in the brain that is required for normal neurodevelopment. More than 300 distinct *MeCP2* loss-of-function mutations are involved in RTT [3]. Other genes, such as *CDKL5* and *FOXG1*, are associated with atypical RTT or RTT-like phenotypes and may convey less severe forms of the disorder.

RTT is the second most frequent genetic cause of intellectual disability in females after Down syndrome [4], with an incidence of 1/10,000 in females and a prevalence of 1:10,000 to 1:22,000 [5]. De novo mutations in the *MeCP2* gene are responsible for RTT, precluding prenatal diagnosis and genetic counseling opportunities. The syndrome is characterized by a period of normal development up to 8 to 30 months of age, followed by an initial plateau in development and subsequent psychomotor regression with loss of acquired skills [6], deceleration of head circumference growth, and development of distinctive repetitive, purposeless hand movements, highly representative of the disorder [7]. Other clinical features include epilepsy, cognitive impairment, scoliosis, feeding difficulties, growth failure, sleep disturbances, bruxism, and motor dysfunctions. Despite the involvement of a single-gene defect, classic RTT exhibits a variety of clinical features with high phenotypic heterogeneity. Indeed, in addition to typical or classical RTT, there are also atypical presentations of the syndrome involving several but not all RTT diagnostic criteria [4]. Formal criteria for the diagnosis of atypical RTT have been defined to encompass cases that vary in terms of age of onset (congenital and early onset seizure variants) and symptom severity (preserved speech and forme fruste variants). Recently, some authors described mutations of *CDKL5* in early seizure variants and of *FOXG1* in congenital variant cases.

Despite dominant neurological manifestations involving tonus abnormalities, motor and language apraxia, and seizures, the disease extends beyond the central nervous system (CNS), affecting a diverse range of non-neurological organs. Multisystemic comorbidities (Table 1), including gastrointestinal, orthopedic, endocrine, or cardiac concerns, exhibit variable prevalence [8]. Frequent symptoms are also waking respiratory irregularities, constipation, and peripheral vasomotor disturbances.

Systematic data regarding endocrine disorders in RTT are still limited and not univocal. Although endocrine disorders seem to be less common than other comorbidities, they are still more frequent than those in the general population. Nevertheless, little is known about endocrinopathies in these patients. Overall, RTT may have a negative impact on growth, gonads, bone health, and the thyroid.

Some authors have reported low bone mineral content as the most common endocrine disorder in RTT [9], followed by alterations in the timing of pubertal onset and menarche [10].

Thyroid function has been rarely studied in RTT patients, with discordant results, even though it is an issue of great concern considering the effect of thyroid hormones on proper mammalian brain development [11].

This study aimed to determine the incidence and clinical significance of endocrine disorders in a pediatric RTT cohort, focusing on the genotype-phenotype correlation.

## 2. Materials and Methods

In this double-center retrospective observational study, patients were enrolled according to the following inclusion criteria: chronological age between 0 and 18 years, Caucasian ethnicity, and genetically confirmed diagnosis of RTT (by sequencing and MLPA). Data were retrospectively collected from the clinical records of patients referred for endocrinological evaluation from the Rett Center of two Italian hospitals (University Hospital “G. Martino”, Messina, Italy; “A. Meyer” Children’s University Hospital, Florence, Italy).

All the patients underwent a comprehensive personal medical history, including data on neurological disorders, autoimmune comorbidities, and current therapies. Clinical, biochemical, and instrumental assessments were also performed, as detailed below.

### 2.1. Clinical Evaluation

The auxological assessment was based on height evaluation and BMI calculations. Physical examination was performed by a dedicated team of pediatric endocrinologists, including measurement of height, weight, BMI, waist circumference (WC), WC-to-height ratio (WHtR), and systolic and diastolic blood pressure. Standing height was measured using a Harpenden stadiometer (Holtain Ltd., Crymych, Dyfed, UK). BMI was calculated using the following equation: body weight(kg)/height(m)^2^. To allow the comparison between different ages and genders, height and BMI were expressed as standard deviation scores (SDS). The pubertal stage was assessed according to the Tanner classification [12].

Obesity was defined as a BMI ≥ +2.0 standard deviation score (SDS) from 5 years and BMI ≥ +3.0 from 0 to 5 years, according to the definition of obesity proposed by the World Health Organization (WHO) for children from the age of 5 years. Malnutrition was defined as a BMI < −2 SDS. Short stature was defined as a height < −2.SDS.

Primary amenorrhea was defined as the absence of menarche by 15 years of age in patients with normal growth and secondary sexual characteristics; secondary amenorrhea was defined as the absence of menses for 3 months in patients with regular menstrual cycles or for ≥6 months in patients with irregular menses; oligo-amenorrhea included ≥3 missed menstrual periods in the preceding six months, and polymenorrhea when menstrual cycles were shorter than 21 days [13].

Pubertal onset was defined as the Tanner B2 stage for females or a testicular volume of more than 4 mL for males (G2).

### 2.2. Biochemical and Instrumental Evaluation

All patients underwent fasting biochemical assessment, including blood sampling for lipid profile, kidney and liver function, electrolytes, blood glucose, insulin, thyroid function, IGF-1, prolactin, ACTH, and cortisol. In postpubertal girls, gonadal function was assessed by measuring gonadotropin and estradiol levels. Screening for celiac disease was performed by measuring the serum transglutaminase-IgA levels. Calcium-phosphorus metabolism was also investigated by measuring the plasma levels of calcium, phosphorus, alkaline phosphatase (ALP), PTH, and cholecalciferol (vitamin D3).

Thyroid ultrasonography (US) was routinely performed. Pelvic ultrasonography was performed in postpubertal girls. Midline defects were excluded using brain MRI. Dual-energy X-ray absorptiometry (DXA) scans were used to measure bone mineral density (BMD). Osteopenia and osteoporosis were defined as reduced BMD Z-scores of <−1 and <−2, respectively. Vitamin D deficiency was defined as a 25-hydroxy vitamin D level of <30 µg/L.

### 2.3. Statistical Analysis

Statistical analyses were performed using IBM SPSS for Windows, Version 22 (IBM Corp., Armonk, NY, USA). A *p*-value lower than 0.05 was considered statistically significant.

Numerical data are expressed as mean, SDS, and range, and categorical variables are expressed as absolute frequencies and percentages. A non-parametric approach was used because the numerical variables were not normally distributed, as verified by the Kolmogorov–Smirnov test.

The Mann–Whitney test was applied with reference to numerical parameters to identify possible significant differences.

The Chi-Square test or exact Fisher test was used to compare differences between the groups with reference to categorical variables (presence or absence of endocrinopathies; presence or absence of epilepsy).

## 3. Results

### 3.1. Main Clinical Features of the Study Population

Fifty-one Caucasian patients (47 girls and 4 boys) with genetically confirmed diagnoses of RTT were enrolled according to the inclusion criteria mentioned above. The mean age of the study population was 9.65 ± 5.9 years (range: 1–18 years).

The main clinical features of the study population are reported in Table 2.

Overall, 37 subjects (72.5%) suffered from epilepsy; 10 of them were diagnosed with drug-resistant epilepsy and 8 with epileptic encephalopathy. Generally, as reported in the literature, epileptic seizures are commonly of multiple types, including complex partial, atypical absence, and generalised tonic–clonic. In RTT, due to *MeCP2* mutation, most patients had multiple epileptic seizure types, although generalised tonic–clonic seizures were the most common. There were no significant clinical differences between the genotypes. 73% of patients with *CDKL5* mutations showed drug-resistant epileptic encephalopathy. In the entire study population, 40 patients required treatment with anti-seizure medications. The main drugs used in these patients were valproic acid (59.4%), carbamazepine (27%), and topiramate (24.3%).

All patients recruited were able to eat solid or semi-solid foods; none of them required percutaneous endoscopic gastrostomy (PEG).

### 3.2. Endocrinological Report

**Figure 1:**
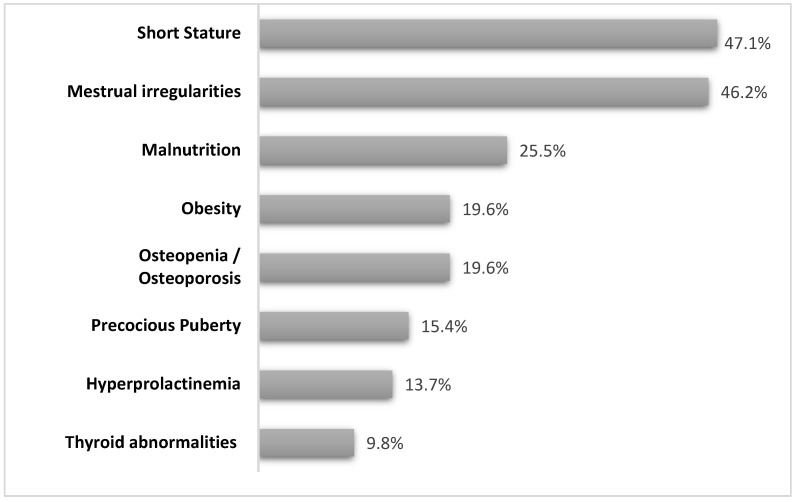
The bar graphic shows the prevalence of endocrine disorders detected in the pediatric population of patients with Rett syndrome.

Overall, 38 patients (74.5%) received one or multiple endocrinological diagnoses and therefore required a dedicated follow-up. The main endocrinological features of the study population are detailed below and summarized in Figure 1 and Table 3.

#### 3.2.1. Short Stature

Twenty-four patients out of 51 (47%) had a height < −2 standard deviation score SDs (mean SDs −3.18, range: −8.26/−2.0) and below parental target height. 7 girls in this group also had BMI < −2 SDs and 4 girls had thyroid dysfunction. Patients with celiac disease and GH deficits were excluded.

#### 3.2.2. Weight Disorders

Thirteen patients (25.5%) were severely underweight (mean BMI SDs −3.79, range: −2.54/−8.19 SDs) in the absence of other chronic diseases that could have caused malabsorption (e.g., celiac disease, inflammatory bowel diseases).

Overall, 10 patients (19.6%) had BMI > 2 SDs (mean BMI +2.35 SDs, range: +2.0/+2.98 SDs). All of them had normal fasting glycemia and lipid profiles; in 2 out of 10 cases, a condition of hyperinsulinemia (fasting insulin > 15 mUI/mL) was diagnosed.

#### 3.2.3. Gonadal Function

Overall, 26 patients were in pubertal age (Tanner stage ≥ B2 for females or G2 for males) in the study population. Among them, 4 girls (15.4%) had a history of precocious puberty, and one girl had a premature isolated pubarche.

Menstrual cycle abnormalities, in particular secondary amenorrhea and oligomenorrhea, were reported in 12/26 (46.1%) patients. Premature ovarian failure (POF) was suspected in two cases, characterized by secondary amenorrhea associated with high levels of gonadotropins (FSH > 25 IU/L). The entire group of patients reported body weight alterations (7 subjects were overweight or obese, and 5 were underweight).

#### 3.2.4. Thyroid Disorders

Thyroid abnormalities were reported in 5 patients (9.8%) among the whole study population. In particular, the main disorders reported were central hypothyroidism (3 patients), subclinical hypothyroidism (1 patient), and Hashimoto’s thyroiditis in the euthyroid phase (1 patient). Two patients in this group also exhibited thyroid nodules on ultrasonography.

#### 3.2.5. Hyperprolactinemia

Serum levels of prolactin above the reference range for sex and age were reported in 7 patients (13.7%), with the occurrence of galactorrhea in one case during anti-seizure therapy. All patients received anti-seizure medications during hormonal evaluation.

#### 3.2.6. Bone Health and Orthopedic Issues

Overall, 11 patients (21.6%) had vitamin D deficiency and therefore required supplementation. DEXA highlighted a picture of reduced bone mineral density in 10 patients: osteopenia was diagnosed in 4 patients, and osteoporosis in 6 patients. Therapy with bisphosphonates was started in two of these patients.

In the whole study population, 17 subjects (33.3%) had moderate-to-severe scoliosis, with surgical treatment required in one case to prevent neurological compromise.

### 3.3. Genotype-Phenotype Correlation

*MeCP2* mutations were detected in 38 patients (74.5%), *CDKL5* deletions in 11 patients (21.6%), and 2 patients (3.9%) exhibited *FOXG1* mutations.

**Figure 2:**
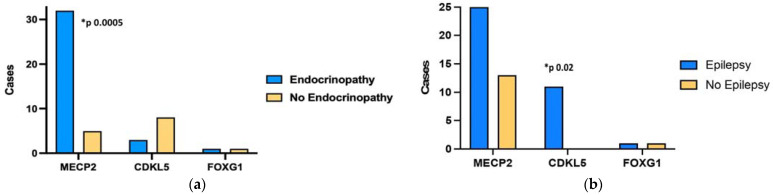
Prevalence of endocrinopathies (**a**) and epilepsy (**b**) and their association with genotypes in a pediatric population of Rett syndrome patients.

In the entire study population, endocrinopathies were significantly more frequent in patients with *MeCP2* mutations (*p* = 0.0005), as shown in Figure 2a. Nevertheless, none of the endocrinopathies mentioned above were significantly associated with a specific genotype. Epilepsy was found to be more frequent in patients with *CDKL5* deletions (*p* = 0.02), as shown in Figure 2b.

## 4. Discussion

RTT is the second most prevalent genetic cause of intellectual disability in girls [4]. Although neurological symptoms are predominant, other organs and apparatus can be affected [14] because of the ubiquitous expression of the *MeCP2* protein. The endocrine system is often involved in a broad spectrum of disorders.

Nevertheless, to the best of our knowledge, data on endocrinopathies in RTT are scarce and not univocal. In the present study, we reported the prevalence of endocrine disorders detected in a pediatric population of RTT patients. In the entire cohort, 74.5% of patients received one or multiple diagnoses necessitating a specific endocrinological follow-up. This finding highlights a prevalence similar to the few literature reports available, especially for short stature, bone alteration, and thyroid disorders [8,9,14,15]. The most frequent endocrinopathy in our RTT population was short stature (47%), followed by menstrual cycle abnormalities (46.2%) and weight disorders (45.1%). Growth failure is one of the supportive diagnostic criteria of RTT [4] and was first described in RTT patients by Schultz et al., usually starting with a deceleration of linear growth velocity by the age of 16 months [16]. Growth studies on RTT have been limited to small populations [17,18]. Huppke et al. showed no evidence that growth retardation in RTT is caused by growth hormone deficiency. Nevertheless, dysfunctional hypothalamic control cannot be excluded [19,20]. Interestingly, short stature has been correlated with specific genotypes. Neul et al. (2021) demonstrated a more pronounced growth deficit in patients with truncating mutations, including pre-C-terminal truncation and R270X, compared to missense mutations like R306C, R133C, and C-terminal truncation, which correlated with milder clinical phenotypes [21]. In our RTT population, all patients with short stature exhibited *MeCP2* mutations (Table 3) without predominance of a specific genotype. In this regard, the study from Huppke et al. showed that female RTT patients with *MeCP2* mutations may have smaller occipitofrontal circumference, shorter length, and lower weight at birth, hypothesizing that defects in *MeCP2* could affect intrauterine somatic growth [22]. Furthermore, poor nutritional status could negatively influence growth, mainly because of a decline in feeding abilities that may occur during RTT. Notably, almost 30% of our RTT population with short stature also had a BMI < −2 SDs. In addition to short stature, *malnutrition* is often present in RTT patients in the first few years after the onset of neurological regression. Studies on weight and nutrition revealed that in RTT, underweight could be the consequence of oral-motor dysfunctions and poor self-feeding abilities [17]. For this reason, active nutritional management is encouraged to give RTT patients the chance to reach their growth potential [23]. Some authors have demonstrated that growth failure may also be strongly associated with the clinical severity of the disease (age of onset, dystonia, deambulation, hand use, and language impairment) [18,24], as well as with several comorbidities that could have an impact on growth, such as oropharyngeal and gastrointestinal dysfunction, scoliosis, seizures, and osteopenia [25].

Interestingly, our data showed that weight disorders, both underweight and overweight, seem to be very well represented in RTT. Indeed, besides malnutrition, even *obesity* was one of the major endocrinological issues detected in our RTT patients. However, to the best of our knowledge, little is known about the onset of obesity in patients with RTT. In our study population, the prevalence of obesity was 19.6%, which was higher than that previously reported [26]. Even if the underlying mechanisms of this association have not been elucidated yet, some authors have reported significantly higher leptin levels in RTT patients than in controls, but this finding was not always associated with obesity [27]. Therefore, it was hypothesized that in RTT patients, leptin might be related to factors other than weight balance, such as neuroendocrine and immune regulation [28]. In addition, experimental evidence suggests that *MeCP2* deletion triggers dysregulation of lipid metabolism, characterized by significant upregulation of lipogenic enzyme gene expression and altered hypothalamic feeding regulatory gene expression. In this regard, Wang et al. enhanced the pivotal role of *MeCP2* in pro-opiomelanocortin (POMC) neurons for energy homeostasis regulation, at least in animal models [29].

Even *menstrual cycle irregularities* were a common finding in our study group, predominantly oligomenorrhea and secondary amenorrhea. To date, literature data on menstrual disorders in RTT are scarce [30,31]. Recently, Humphrey et al. described the features of menstruation in 77 RTT females, focusing on dysmenorrhea and emotional lability as common issues and enhancing an increase in catamenial seizure activity in these patients [31], which we did not find in our cohort. Notably, all our RTT patients with menstrual irregularities also suffered from body weight alterations (both overweight and underweight). In addition, hypergonadotropic hypogonadism was diagnosed in two patients with secondary amenorrhea; therefore, POF was suspected based on clinical and laboratory data. The association between RTT and POF remains unknown. It should be considered that *FMR1* (fragile X mental retardation 1) and *FMR2* genes, often involved in POF with genetic etiology, are mapped on the long arm of chromosome X (Xq27.3 and Xq28, respectively), next to *MeCP2* [32]. Furthermore, recent studies have demonstrated that *MeCP2* co-localizes with GnRH within GnRH neurons in the hypothalamus [33,34]. It seems that *MeCP2* could be a potential player in the regulation of human pubertal timing. In this regard, some authors have reported rare heterozygous *MeCP2* mutations in girls with central precious puberty (CPP), with or without neurodevelopmental abnormalities [33]. Of course, early pubertal development has been documented among RTT children due to MeCP2 mutations [10,35,36]. Killian et al. described early thelarche in 25% and early pubarche in 28% of RTT girls, with earlier occurrence of menarche in girls with milder mutations. Likewise, in our RTT population, we identified four girls with precocious puberty and one with early pubarche; in this subgroup of patients, *MeCP2* mutations were highly predominant, as expected (Table 3).

As far as *thyroid function* is concerned, many genetic syndromes are known to be associated with thyroid disorders [37], even though reports on RTT are still limited. In this regard, our data are consistent with the previous findings. In the present study, thyroid disorders accounted for approximately 10%, ranging from autoimmune thyroiditis to central hypothyroidism and hyperthyrotropinemia. We also detected thyroid nodules in two patients. Recently, an Italian clinical trial highlighted significantly higher FT4 levels in RTT patients than in controls, especially in those with *CDKL5* deletions [15]. This finding may reflect the upregulation of TSH signaling within the CNS, thus leading to a compensatory mechanism in response to *MeCP2* dysfunction, considering its established role in neuronal maturation and maintenance [15,38]. In recent years, some authors have hypothesized a possible association between thyroid function and *MeCP2* mutations, suggesting that thyroid hormones may play a pivotal role in the expression of *MeCP2* [39]. Conversely, *MeCP2* dysfunction, characterized by disrupted neuronal maturation and maintenance, leads to aberrant gene expression profiles, potentially contributing to imbalances in excitatory and inhibitory synaptic signaling [38].

Finally, *bone health* issues are relevant in RTT for both motor impairment and skeletal abnormalities. Notably, osteopenia and osteoporosis were found in approximately 20% of our cohort. Previous studies in RTT females showed impaired bone health with decreased mineral content, decreased mineral density, and an increased fracture rate three to four times—than controls, reporting a higher prevalence of osteopenia and osteoporosis compared to our study, probably due to the limited availability of testing outside of the research setting or particular clinical situation. Orthopedic problems, including scoliosis and joint contractures, are also frequent [40]. Scoliosis is related to the lack of walking but is unrelated to the loss of hand skills or hand stereotypes. Such alterations in bone mineral deposition may be caused by vitamin D deficiency, which is typical of the RTT population [41]; therefore, adequate supplementation is required in these patients. Interestingly, Jefferson et al. highlighted that lower bone density in RTT may be directly associated with the type of *MeCP2* mutation [42]. Indeed, a lack of *MeCP2* might reduce bone density through osteoblastic dysfunction [43]. However, a better understanding of the molecular mechanisms underlying RTT bone problems is needed to allow new potential pharmacological strategies and targets for these complications that adversely affect the quality of life in RTT individuals from a younger age.

Interestingly, in addition to RTT, endocrinopathies seem to be common in other genetic disorders causing intellectual impairment, such as Down syndrome, Williams syndrome, and Prader-Willi syndrome. For example, patients with Down syndrome are at a higher risk of developing thyroid disorders, type 1 diabetes mellitus, short stature, obesity, gonadal dysfunction, and vitamin D deficiency [37,44]. Similarly, Williams-Beuren syndrome is often characterized by growth restriction, overweight, precocious puberty, and thyroid dysfunction [45]. In Prader-Willi syndrome, obesity is often associated with other hypothalamic dysfunctions, including GH deficiency, hypogonadism, hypothyroidism, and adrenal insufficiency [46].

Overall, to the best of our knowledge, the present study highlighted for the first time that RTT patients with *MeCP2* deletions seem to be exposed to a higher risk of developing endocrinopathies, regardless of the type of gland or apparatus involved. In addition, our study showed that the *CDKL5* genotype was more frequently associated with epilepsy, which is in accordance with other studies. These data are not clearly reported in the literature, maybe because epileptic disorders are often present in RTT individuals, especially with the classical form associated with *MeCP2* deletion. However, *CDKL5* mutations have been described in early-onset seizure variants of RTT and early-onset epileptic encephalopathies [47]. However, the effects of antiepileptic drugs on thyroid function and bone and metabolic health need to be considered in the interpretation of our results. More studies are necessary to assess this issue. Furthermore, due to the dicentric nature of the present study, any differences in the management and follow-up of the patients could have partially influenced the results.

In addition to the available literature reports, our results support the need for endocrinological evaluation and follow-up in RTT patients to prevent and detect endocrinological comorbidities at an early stage. A proposal for RTT patients’ endocrinological management is presented in Table 4.

We acknowledge that our study has some limitations, above all, a relatively small population, even if counterbalanced by the rarity of the disease.

Future studies on larger RTT populations are needed to confirm and clarify this genotype-phenotype correlation, which could become predictive value, helping to identify patients at increased risk at an early stage.

## 5. Conclusions

RTT is a severe neurological disorder that has increasingly emerged in recent years. Multiple organs and apparatuses can be involved with a broad spectrum of manifestations. The present study proves that endocrinopathies are not rare in RTT patients, with short stature, menstrual cycle abnormalities, and weight disorders being the most frequent endocrinological reports. Interestingly, patients with *MeCP2* deletions seemed to be at a higher risk for developing endocrinopathies.

Future studies with larger cohorts could confirm our findings, helping identify effective screening strategies and new therapeutic choices.

Therefore, in the context of a multidisciplinary approach, endocrinological evaluation and follow-up should be recommended in RTT patients to prevent and detect endocrinological comorbidities at an early stage and improve their quality of life.

## Figures and Tables

**Table 1:** Rett syndrome-related organ system disorders.

Organ/System	Disorders/Abnormalities
Respiratory system	Breathing arrhythmias (apneas) Breath-holding
Cardiovascular system	Rythm defects QT prolonged interval No structural changes
Digestive system	Gastrointestinal dysmotility (Dysphagia) Altered microbiota
Metabolic system	Severe dyslipidemia Fatty liver disease Metabolic syndrome Insulin resistance Alters energy homeostasis
Skeletal system	Scoliosis Fractures
Endocrine system	Low-bone mineral content Delayed menarche Thyroid hormones
Muscular system	Mild hypotonia Muscle atrophy
Urinary system	Urinary tract infection Kidney stones Urine retention

**Table 2:** Main clinical and genetic features of the study population.

Patients (n)	51
Males, n (%)	4 (7.8%)
Females, n (%)	47 (92.2%)
Age at visit (mean, SDS)	9.65 ± 5.9
Age at diagnosis (mean, SDS)	3.22 ± 2.4
Height (mean, SDS)	−1.76 ± 1.84
BMI (mean, SDS)	−0.77 ± 2.53
Prepubertal, n (%)	25 (49%)
Pubertal, n (%)	26 (51%)
Genotype	
*MeCP2*	38 (74.5%)
*CDKL5*	11 (21.6%)
*FOXG1*	2 (3.9%)
Epilepsy	37 (72.5%)
Drug-resistant epilepsy	10 (27%)
Epileptic encephalopathy	8 (21.6%)

Body mass index (BMI), standard deviation score (SDS). Numerical data are expressed as mean ± SDS.

**Table 3:** Genotypic and endocrinological features in a cohort of Rett syndrome patients.

Patient n.	Gene	Mutation/Deletion	Endocrine Disease
1	*MeCP2*	P322A	Obesity, amenorrhea (POF), osteopenia, vitamin D deficiency
2	*MeCP2*	P322S	None
3	*MeCP2*	R133C	Short stature, obesity, amenorrhea (POF), thyroid nodules, hyperprolactinemia, osteopenia
4	*MeCP2*	R133C	Obesity
5	*MeCP2*	R168X	Short stature, obesity, menstrual irregularities, central hypothyroidism, hyperprolactinemia, osteoporosis
6	*MeCP2*	R168X	Short stature, precocious puberty
7	*MeCP2*	R255X	Short stature, malnutrition
8	*MeCP2*	R255X	Short stature, malnutrition, menstrual irregularities, hyperprolactinemia, central hypothyroidism, thyroid nodules
9	*MeCP2*	R255X	Short stature, precocious puberty
10	*MeCP2*	R270X	Short stature, osteoporosis, vitamin D deficiency
11	*MeCP2*	R270X	Short stature, malnutrition, menstrual irregularities
12	*MeCP2*	R270X	Premature pubarche, vitamin D deficiency
13	*MeCP2*	R294X	vitamin D deficiency
14	*MeCP2*	R294X	Short stature, vitamin D deficiency
15	*MeCP2*	R294X	Malnutrition, osteopenia
16	*MeCP2*	R294X	Malnutrition
17	*MeCP2*	R306C	Short stature, obesity, menstrual irregularities, Hashimoto’s thyroiditis, hyperprolactinemia
18	*MeCP2*	R306C	Short stature, malnutrition, menstrual irregularities, hyperprolactinemia
19	*MeCP2*	R306C	Short stature, hyperprolactinemia
20	*MeCP2*	R306X	Short stature, precocious puberty
21	*MeCP2*	S134C	Short stature, obesity, menstrual irregularities, subclinical hypothyroidism, vitamin D deficiency
22	*MeCP2*	T158M	Short stature, overweight, osteopenia
23	*MeCP2*	T158M	Short stature, malnutrition, menstrual irregularities, osteoporosis, vitamin D deficiency
24	*MeCP2*	T158M	Malnutrition
25	*MeCP2*	T158P	Osteoporosis
26	*MeCP2*	T158M	Short stature, malnutrition, menstrual irregularities, central hypothyroidism, vitamin D deficiency
27	*MeCP2*	c.749_752dup(p.Gly252Profs * 8)	Obesity
28	*MeCP2*	c.763C>T	Short stature, osteoporosis
29	*MeCP2*	c.880C>T	Short stature, osteoporosis
30	*MeCP2*	c.880>T	Short stature, malnutrition
31	*MeCP2*	c.880>T p, (Arg294 *)	Short stature
32	*MeCP2*	c.316C>T (p.Arg106Trp)	Short stature, vitamin D deficiency
33	*MeCP2*	c.915G>T (p.Lys305Asn)	Obesity, menstrual irregularities
34	*MeCP2*	c.917G>A	Obesity
35	*MECP2*	c.1061_1062del p.(Arg354Glnfs * 38)	Short stature, malnutrition
36	*MeCP2*	1097 delinsCC; 1130_1190delinG *	Short stature
37	*MeCP2*	c.1164_1207del [p.(Pro389 *)	Obesity, menstrual irregularities, hyperprolactinemia, vitamin D deficiency
38	*MeCP2*	c.1164_1207del [p.(Pro389 *)	None
39	*CDKL5*	whole gene del	None
40	*CDKL5*	whole gene del	Precocious puberty
41	*CDKL5*	c.119 C>T (p.Ala40Val)	None
42	*CDKL5*	c.119 C>T (p.Ala40Val)	Malnutrition, vitamin D deficiency
43	*CDKL5*	c.380A>G [p.his127Arg]	None
44	*CDKL5*	c628G>A p (Gly228Arg)	None
45	*CDKL5*	Exon 1	None
46	*CDKL5*	c.855A>T; p Arg 285 ser	None
47	*CDKL5*	c.1648C>T p.(Arg550 *)	None
48	*CDKL5*	c.2217dup p.(Pro740Thrfs * 24)	None
49	*CDKL5*	c.2713+19G>A e c.2732G>A	None
50	*FOXG1*	C.256delC	None
51	*FOXG1*	c.681C>G	Malnutrition

* Double heterozygous variants. POF (premature ovarian failure).

**Table 4:** Proposed endocrinological evaluation for Rett Syndrome patients in clinical practice across different age ranges.

Endocrinological Evaluation	Infancy	Childhood-Adolescence	Adulthood
***Growth assessment***			
*Height*	√	√	NA
*Growth velocity*	√	√	NA
*Weight, BMI*	√	√	√
*IGF-1 and GH secretion*	in case of short stature and/or growth deceleration	in case of short stature and/or growth deceleration	NA
*Bone age*	√	√	NA
***Thyroid function***			
*FT4, TSH, TPO-AB, TG-AB*	√	√	√
*Thyroid ultrasound*	if thyroid alterations, goitre or family history	if thyroid alterations, goitre or family history	if thyroid alterations, goitre or family history
***Metabolic assessment***			
*Blood pressure*	√	√	√
*Waist circumference*	if BMI > 2 SDS	if BMI > 2 SDS	if BMI > 2 SDS
*Blood glucose, Hba1c*	√	√	√
*Insulin*	if BMI > 2 SDS	if BMI > 2 SDS	if BMI > 2 SDS
*Lipid profile*	√	√	√
***Gonadal function***			
*Gonadotropins*	NA	in case of pubertal alterations or menstrual irregularities	in case of menstrual irregularities
*Sex steroids*	NA	in case of pubertal alterations or menstrual irregularities	in case of menstrual irregularities
***Bone health***			
*Calcium-phosphorus metabolism*	√	√	√
*DEXA*	NA	√	√

BMI, body mass index; SDS, standard deviation score; DEXA, dual-energy X-ray absorptiometry. √, to be performed at diagnosis and periodically thereafter.

## Data Availability

All data generated or analyzed during this study are available from the corresponding author upon request.

## References

[1] Iourov I.Y., Vorsanova S.G., Voinova V.Y., Kurinnaia O.S., Zelenova M.A., Demidova I.A., Yurov Y.B. (2013). Xq28 (MECP2) Microdeletions Are Common in Mutation-Negative Females with Rett Syndrome and Cause Mild Subtypes of the Disease. Mol. Cytogenet..

[2] Amir R.E., Van den Veyver I.B., Wan M., Tran C.Q., Francke U., Zoghbi H.Y. (1999). Rett Syndrome Is Caused by Mutations in X-Linked MECP2, Encoding Methyl-CpG-Binding Protein 2. Nat. Genet..

[3] Tillotson R., Bird A. (2020). The Molecular Basis of MeCP2 Function in the Brain. J. Mol. Biol..

[4] Neul J.L., Kaufmann W.E., Glaze D.G., Christodoulou J., Clarke A.J., Bahi-Buisson N., Leonard H., Bailey M.E., Schanen N.C., Zappella M. (2010). Rett Syndrome: Revised Diagnostic Criteria and Nomenclature. Ann. Neurol..

[5] Percy A.K. (2002). Rett Syndrome: Current Status and New Vistas. Neurol. Clin..

[6] Hagberg B., Aicardi J., Dias K., Ramos O. (1983). A Progressive Syndrome of Autism, Dementia, Ataxia, and Loss of Purposeful Hand Use in Girls: Rett’s Syndrome: Report of 35 Cases. Ann. Neurol..

[7] Halbach N.S.J., Smeets E.E.J., Steinbusch C., Maaskant M.A., Van Waardenburg D., Curfs L.M.G. (2013). Aging in Rett Syndrome: A Longitudinal Study. Clin. Genet..

[8] Fu C., Armstrong D., Marsh E., Lieberman D., Motil K., Witt R., Standridge S., Lane J., Dinkel T., Jones M. (2020). Multisystem Comorbidities in Classic Rett Syndrome: A Scoping Review. BMJ Paediatr. Open.

[9] Motil K.J., Ellis K.J., Barrish J.O., Caeg E., Glaze D.G. (2008). Bone Mineral Content and Bone Mineral Density Are Lower in Older than in Younger Females with Rett Syndrome. Pediatr. Res..

[10] Killian J.T., Lane J.B., Cutter G.R., Skinner S.A., Kaufmann W.E., Tarquinio D.C., Glaze D.G., Motil K.J., Neul J.L., Percy A.K. (2014). Pubertal Development in Rett Syndrome Deviates from Typical Females. Pediatr. Neurol..

[11] Thompson C.C., Potter G.B. (2000). Thyroid Hormone Action in Neural Development. Cereb. Cortex.

[12] Tanner J.M. (2009). Growth and Maturation during Adolescence. Nutr. Rev..

[13] American Academy of Pediatrics, Committee on Adolescence, American College of Obstetricians and Gynecologists (2006). Committee on Adolescent Health Care Menstruation in Girls and Adolescents: Using the Menstrual Cycle as a Vital Sign. Pediatrics.

[14] Borloz E., Villard L., Roux J.-C. (2021). Rett Syndrome: Think Outside the (Skull) Box. Fac. Rev..

[15] Stagi S., Giani T., Simonini G., Falcini F. (2005). Thyroid Function, Autoimmune Thyroiditis and Coeliac Disease in Juvenile Idiopathic Arthritis. Rheumatology.

[16] Schultz R.J., Glaze D.G., Motil K.J., Armstrong D.D., del Junco D.J., Hubbard C.R., Percy A.K. (1993). The Pattern of Growth Failure in Rett Syndrome. Am. J. Dis. Child.

[17] Thommessen M., Kase B., Heiberg A. (1992). Growth and Nutrition in 10 Girls with Rett Syndrome. Acta Paediatr..

[18] Cass S.R. (2001). Hilary Growth and Nutrition in Rett Syndrome. Disabil. Rehabil..

[19] Huppke P., Roth C., Christen H., Brockmann K., Hanefeld F. (2001). Endocrinological Study on Growth Retardation in Rett Syndrome. Acta Paediatr..

[20] Hara M., Nishi Y., Yamashita Y., Hirata R., Takahashi S., Nagamitsu S., Hosoda H., Kangawa K., Kojima M., Matsuishi T. (2014). Relation between Circulating Levels of GH, IGF-1, Ghrelin and Somatic Growth in Rett Syndrome. Brain Dev..

[21] Neul J.L., Fang P., Barrish J., Lane J., Caeg E.B., Smith E.O., Zoghbi H., Percy A., Glaze D.G. (2008). Specific Mutations in *Methyl-CpG-Binding Protein 2* Confer Different Severity in Rett Syndrome. Neurology.

[22] Huppke P., Held M., Laccone F., Hanefeld F. (2003). The Spectrum of Phenotypes in Females with Rett Syndrome. Brain Dev..

[23] Oddy W.H., Webb K.G., Baikie G., Thompson S.M., Reilly S., Fyfe S.D., Young D., Anderson A.M., Leonard H. (2007). Feeding Experiences and Growth Status in a Rett Syndrome Population. J. Pediatr. Gastroenterol. Nutr..

[24] Wong L.C., Chen Y., Tsai S., Lin Y., Hsu C., Wang H., Hu S., Shen H., Tsai W., Lee W. (2021). Dietary Intake and Growth Deficits in Rett Syndrome—A Cross-section Study. Autism Res..

[25] Tarquinio D.C. (2012). Growth Failure and Outcome in Rett Syndrome. Neurology.

[26] Motil K.J., Caeg E., Barrish J.O., Geerts S., Lane J.B., Percy A.K., Annese F., McNair L., Skinner S.A., Lee H.-S. (2012). Gastrointestinal and Nutritional Problems Occur Frequently throughout Life in Girls and Women with Rett Syndrome. J. Pediatr. Gastroenterol. Nutr..

[27] Blardi P., De Lalla A., D’Ambrogio T., Zappella M., Cevenini G., Ceccatelli L., Auteri A., Hayek J. (2007). Rett Syndrome and Plasma Leptin Levels. J. Pediatr..

[28] Bornstein S.R., Licinio J., Tauchnitz R., Engelmann L., Negrão A., Gold P., Chrousos G.P. (1998). Plasma Leptin Levels Are Increased in Survivors of Acute Sepsis: Associated Loss of Diurnal Rhythm in Cortisol and Leptin Secretion. J. Clin. Endocrinol. Metab..

[29] Wang X., Lacza Z., Sun Y.E., Han W. (2014). Leptin Resistance and Obesity in Mice with Deletion of Methyl-CpG-Binding Protein 2 (MeCP2) in Hypothalamic pro-Opiomelanocortin (POMC) Neurons. Diabetologia.

[30] Quint E.H. (2008). Menstrual Issues in Adolescents with Physical and Developmental Disabilities. Ann. N. Y. Acad. Sci..

[31] Humphrey K.N., Horn P.S., Olshavsky L., Reebals L., Standridge S.M. (2021). Features of Menstruation and Menstruation Management in Individuals with Rett Syndrome. J. Pediatr. Adolesc. Gynecol..

[32] Ferreira S.I., Matoso E., Pinto M., Almeida J., Liehr T., Melo J.B., Carreira I.M. (2010). X-Chromosome Terminal Deletion in a Female with Premature Ovarian Failure: Haploinsufficiency of X-Linked Genes as a Possible Explanation. Mol. Cytogenet..

[33] Canton A.P.M., Tinano F.R., Guasti L., Montenegro L.R., Ryan F., Shears D., De Melo M.E., Gomes L.G., Piana M.P., Brauner R. (2023). Rare Variants in the MECP2 Gene in Girls with Central Precocious Puberty: A Translational Cohort Study. Lancet Diabetes Endocrinol..

[34] Huang Q., Gong Y., Liu H., Zhao J., Wang J., Lu W. (2021). Expression and Distribution Pattern of DNMT1 and MeCP2 and Their Relationship with GnRH and Kisspeptin in the Hypothalamus during Puberty Onset in Ewes. IJAR.

[35] Garcia-Rudaz C., Deng V., Matagne V., Ronnekleiv O.K., Bosch M., Han V., Percy A.K., Ojeda S.R. (2009). FXYD1, a Modulator of Na^+^, K^+^ -ATPase Activity, Facilitates Female Sexual Development by Maintaining Gonadotrophin-Releasing Hormone Neuronal Excitability. J. Neuroendocrinol..

[36] Yang L., Jiang M., Yu R., Hu R., Xiong F., Li J. (2021). A Case Report of Precocious Puberty Related to Rett Syndrome and a Literature Review. Pharmazie.

[37] Casto C., Pepe G., Li Pomi A., Corica D., Aversa T., Wasniewska M. (2021). Hashimoto’s Thyroiditis and Graves’ Disease in Genetic Syndromes in Pediatric Age. Genes.

[38] Kron M., Howell C.J., Adams I.T., Ransbottom M., Christian D., Ogier M., Katz D.M. (2012). Brain Activity Mapping in *Mecp2* Mutant Mice Reveals Functional Deficits in Forebrain Circuits, Including Key Nodes in the Default Mode Network, That Are Reversed with Ketamine Treatment. J. Neurosci..

[39] Bunker S.K., Dandapat J., Chainy G.B.N., Sahoo S.K., Nayak P.K. (2017). Neonatal Exposure to 6-n-Propyl-Thiouracil, an Anti-Thyroid Drug, Alters Expression of Hepatic DNA Methyltransferases, Methyl CpG-Binding Proteins, Gadd45a, P53, and PCNA in Adult Male Rats. Eur. Thyroid. J..

[40] Leonard H., Thomson M., Bower C., Fyfe S., Constantinou J. (1995). Skeletal Abnormalities in Rett Syndrome: Increasing Evidence for Dysmorphogenetic Defects. Am. J. Med. Genet..

[41] Motil K.J., Barrish J.O., Lane J., Geerts S.P., Annese F., McNair L., Percy A.K., Skinner S.A., Neul J.L., Glaze D.G. (2011). Vitamin D Deficiency Is Prevalent in Girls and Women with Rett Syndrome. J. Pediatr. Gastroenterol. Nutr..

[42] Jefferson A.L., Woodhead H.J., Fyfe S., Briody J., Bebbington A., Strauss B.J., Jacoby P., Leonard H. (2011). Bone Mineral Content and Density in Rett Syndrome and Their Contributing Factors. Pediatr. Res..

[43] O’Connor R.D., Zayzafoon M., Farach-Carson M.C., Schanen N.C. (2009). Mecp2 Deficiency Decreases Bone Formation and Reduces Bone Volume in a Rodent Model of Rett Syndrome. Bone.

[44] Metwalley K.A., Farghaly H.S. (2022). Endocrinal Dysfunction in Children with Down Syndrome. Ann. Pediatr. Endocrinol. Metab..

[45] Levy-Shraga Y., Gothelf D., Pinchevski-Kadir S., Katz U., Modan-Moses D. (2018). Endocrine Manifestations in Children with Williams–Beuren Syndrome. Acta Paediatr..

[46] Heksch R., Kamboj M., Anglin K., Obrynba K. (2017). Review of Prader-Willi Syndrome: The Endocrine Approach. Transl. Pediatr..

[47] Artuso R., Mencarelli M.A., Polli R., Sartori S., Ariani F., Pollazzon M., Marozza A., Cilio M.R., Specchio N., Vigevano F. (2010). Early-Onset Seizure Variant of Rett Syndrome: Definition of the Clinical Diagnostic Criteria. Brain Dev..

